# URG11 Regulates Prostate Cancer Cell Proliferation, Migration, and Invasion

**DOI:** 10.1155/2018/4060728

**Published:** 2018-05-31

**Authors:** Bin Pan, Yunlin Ye, Haiping Liu, Jianli Zhen, Hongmei Zhou, Yutong Li, Lijun Qu, Youke Wu, Chuanrong Zeng, Weifeng Zhong

**Affiliations:** ^1^Department of Urology, The First Affiliated Hospital, Jinan University, Guangzhou, China; ^2^Department of Urology, Sun Yat-sen Cancer Center, State Key Laboratory of Oncology in South China, Collaborative Innovation Center of Cancer Medicine, Guangzhou, China; ^3^Department of Surgery, Lianping Second People's Hospital, Heyuan 517139, China; ^4^Department of Surgery and Anesthesia, Sun Yat-sen University Cancer Center, State Key Laboratory of Oncology in South China, Collaborative Innovation Center for Cancer Medicine, Guangzhou, Guangdong 510060, China; ^5^Department of Urology, Meizhou People's Hospital, Meizhou 514031, China

## Abstract

Upregulated gene 11 (URG11), a new gene upregulated by hepatitis B virus X protein, is involved in the development and progression of several tumors, including liver, stomach, lung, and colon cancers. However, the role of URG11 in prostate cancer remains yet to be elucidated. By determined expression in human prostate cancer tissues, URG11 was found significantly upregulated and positively correlated with the severity of prostate cancer, compared with that in benign prostatic hyperplasia tissues. Further, the mRNA and protein levels of URG11 were significantly upregulated in human prostate cancer cell lines (DU145, PC3, and LNCaP), compared with human prostate epithelial cell line (RWPE-1). Moreover, by the application of siRNA against URG11, the proliferation, migration, and invasion of prostate cancer cells were markedly inhibited. Genetic knockdown of URG11 also induced cell cycle arrest at G1/S phase, induced apoptosis, and decreased the expression level of *β*-catenin in prostate cancer cells. Overexpression of URG11 promoted the expression of *β*-catenin, the growth, the migration, and invasion ability of prostate cancer cells. Taken together, this study reveals that URG11 is critical for the proliferation, migration, and invasion in prostate cancer cells, providing the evidence of URG11 to be a novel potential therapeutic target of prostate cancer.

## 1. Introduction

Prostate cancer (PCa) is one of the most common cancers of urinary system and the second most common cause of cancer-related death in men in western world [1]. Metastasis is the leading cause of morbidity and mortality of prostate cancer. Once prostate cancer has metastasized, the disease becomes a terminal disease [2, 3]. Thus development of new therapeutic targets and reliable biomarkers for detection of metastatic potential is of specific importance. Metastasis is a complex multistep process regulated by a variety of genes and signaling pathways. Cytokine induced cancer cell specific oriented homing and increased adhesion opacity to organic microvascular endothelium, tumor angiogenesis and lymphangiogenesis, and other factors combine together to impact the process [4]. Among these factors, epithelial-mesenchymal transition (EMT) is an important event for cancer cell movement, initiating the initial phase of metastasis [5].

Upregulated gene 11 (URG11) is a newly identified gene upregulated by hepatitis B virus X protein, located on the long arm of human chromosome 11, encoding a 70-kDa protein that consists of five chordin-like cysteine-rich repeats and a single C-type lectin domain [6]. These domains are reported to be related to cell adhesion, migration, and cell/matrix interaction [7–9], indicating the important role in cancer cell EMT. Accordingly, URG11 is overexpressed in many cancer cells and tissues and promotes cancer development and progression. Thus, URG11 is reported as a cancer cell regulator. For example, URG11 was highly expressed in pancreatic cancer tissues and inhibition of URG11 by RNA interference regulated the expression of EMT markers and suppressed pancreatic cancer cell invasion [10]. Also, URG11 is reported with gastric cancer development. URG11 was highly expressed in gastric cancer specimens and closely related to TNM stage and lymph node metastasis; URG11 promoted the transition of G1/S stage and increased ability of cell adhesion and invasion; knock down of URG11 significantly downregulated cell proliferation, anchorage-independent growth, and invasion of gastric cancer cells [11].

However, the role of URG11 in human prostate cancer remains to be determined. Therefore, the aim of the present study is to explore the role of URG11 in human prostate cancer, trying to reveal the expression of URG11 in human prostate cancer cells and in clinical human prostate cancer tissues and the relationship of URG11 with cell growth, migration, and invasion. The data demonstrates that URG11 may play a critical role in human prostate cancer progression.

## 2. Methods and Materials

### 2.1. Ethics Statement

All human and animal experiments were approved by the Jinan University. All experiments using human specimens were performed in accordance with the Declaration of Helsinki. Informed consent was received from all participated subjects prior to the study. 68 cases of human tissue samples of prostate cancer and 74 cases of benign prostatic hyperplasia were obtained from prostate patients in the First Affiliated Hospital of Jinan University and Sun Yat-sen University Cancer Center, from Jun 2010 to Jun 2013. All animal procedures followed the humane care guidelines of the Chinese National Institute of Health, and the protocols were approved by the Committee on Animal Research of Jinan University.

### 2.2. Cell Culture

Cell lines human prostate cancer DU145 and LNCaP were from the First Affiliated Hospital of Jinan University, human prostate cancer PC3 cell was a gift from NanFang Hospital of Southern Medical University, and nontumor human prostate epithelial cells (RWPE-1) were a gift from School of Pharmaceutical Sciences of Guangzhou Medical University. Cells were maintained in medium RPMI 1640 (HyClone, Logan, UT, USA) supplemented with 10% fetal bovine serum (FBS, Sigma-Aldrich Chemie, Steinheim, Germany), penicillin (Sigma, 100 U/ml), and streptomycin (Sigma, 100 *μ*g/ml) at 37°C in a humidified atmosphere with 5% CO_2_.

### 2.3. RNA Extraction and Real-Time PCR

Total RNA was isolated using Trizol reagent (Toyobo, Carlsbad, CA) according to the manufacturer's protocol. For real-time quantitative PCR, equal amounts of cDNA were added to SYBR premix EX Taq II (Toyobo) and run in Stepone real-time PCR system (AB Applied Biosystems, Foster City, CA). The cycling program was 95°C for 5 s and 60°C for 30 s. The primers for real-time PCR are URG11 sense 5'-TGAATCAAGGAGTCGCTGGAC-3'; URG11 anti-sense 5'-GCATCTCACTGGAACACAAG-3'; GAPHD sense 5'-CAACGGATTTGGTCGTATTGGG-3'; GAPHD antisense 5'-CCTGGAAGATGGTGATGGGATT -3'. Each sample was assayed in triplicate, and GAPDH was used as an endogenous control. Results were normalization to the expression of GAPDH.

### 2.4. siRNA Design and Transfection

siRNA duplexes (mixture of multiple siRNA fragments) targeting URG11 were synthesized by GenePharm (Shanghai, China). The siRNA sequences against URG11 are CAGACGGAUUGCUGUACUU and ACACAGACUUUACCUACAA [12]. Optimal transfection of prostate control and cancer cells were transfected by Lipofectamine 2000 (Invitrogen, Carlsbad, California, USA) with siRNA fragment at a final concentration of 100 nmol/L. A scrambled siRNA sequence targeting no gene supplied by GenePharm is used as siRNA control (NC).

### 2.5. Western Blotting

Cells were lysed in sample buffer and subjected to sodium dodecyl sulfate-polyacrylamide gel electrophoresis as described previously [13]. Primary antibodies against URG11 and *β*-catenin were obtained from Santa Cruz Biotechnology, Santa Cruz, CA, USA). GAPDH (Santa Cruz) was used as the loading control. Polyvinylidene fluoride (PVDF) membrane (Millipore, Boston, MA, USA) transferred with proteins were washed with PBS containing 0.1% Tween-20 five times, the membrane was incubated with the appropriate horseradish peroxidase-conjugated secondary antibodies (Amersham Biosciences, Uppsala, Sweden) for 1 h, and bands were detected by enhanced chemiluminescence (Amersham, Bucks, UK). Densitometric values were normalized to GAPDH levels.

### 2.6. MTS

MTS assay was applied to detect cancer cell growth as previously reported [13]. Cell proliferation was assessed with the CellTiter 96^®^ AQueous One Solution Cell Proliferation Assay Kit (Promega, Madison, WI, USA) accordingly. In each group, cells were allowed to grow for 6, 24, 48, 72, and 96 h. At harvesting, 20 *μ*l of CellTiter 96 AQueous One Solution reagent was added to each well in a total volume of 100 *μ*l of medium for 3–4 h. Absorbance was measured at 490 nm using an ELISA plate reader. The growth rate was calculated from the absorbance, and the readings at 6 h time points in each group were set to 100%.

### 2.7. Annexin V and Propidium Iodide (PI) Double Staining and FACS Analysis

Annexin V-FITC Apoptosis Detection Kit (BIPEC, USA) was used to determine apoptotic cells and PI staining was used to reveal cell cycle stage. In brief, cells were resuspended with 400 *μ*l binding buffer at 10^6^ cells/ml, labeled with annexin V-FITC for 15 min and with PI for another 5 min; then cells were analysed by flow cytometry by a FACScan flow cytometer (BD Biosciences, Mountain View, CA, USA).

### 2.8. Would Healing Assay

Cells were grown in six-well dishes until confluence; then cell culture was scraped with a pipette tip. Cells were washed twice with PBS and cultured in a 5% CO_2_ incubator for 48 h at 37°C. Images of each well were acquired immediately and at 6, 12, and 24 h in four random fields using an inverted fluorescence microscope (Nikon Corporation, Tokyo, Japan) at ×100 magnification. Wound closure was expressed as the average ± SD of the difference between the measurements at time zero and each time period.

### 2.9. Migration and Invasion Assay

Migration and invasion assays were performed using a Transwell system (Millipore, MA, USA), accordingly. Cells were seeded in the upper chamber of the Transwell insert and incubated with 0.5% DMSO or deguelin (10 and 15 *μ*M), and 90% DMEM containing 10% FBS was added to the lower chamber and incubated for 24 h. The remaining cells in the upper chamber were removed. Cells migrating into the lower surface of the filter were fixed by PFA and stained with 2% crystal violet for quantification. For the invasion assay, the Transwell filter membrane was coated with Matrigel (Becton Dickinson Bioscience, MA, USA). Cell numbers were expressed as the mean ± SD.

### 2.10. Immunohistochemistry

For immunohistochemistry [14], paraffin sections were treated with hydrogen peroxide to inactivate endogenous peroxidases. Antigen retrieval was performed in a microwave in 10 mM citrate buffer at pH 6.0. Sections were fixed with paraformaldehyde followed by permeabilization and blocking. Sections were then incubated in anti-URG11 (Santa Cruz) antibody overnight at 4°C, and a secondary antibody was used to detect protein expression. Immunostaining was analysed with the Super Sensitive Non-Biotin Polymer HRP Detection System according to the manufacturer's instructions (BioGenex, San Ramon, Canada).

For analysis, randomly selected five fields of the staining were captured; the grade values were determined by the percentage of positive stained cells and the strength of URG11 staining [15]. Briefly, the ratio of positive cells scored 0 for staining of ≤1%, 1 for staining of 2 to 25%, 2 for staining of 26 to 50%, 3 for staining of 51 to 75%, and 4 for staining >75%. Staining intensity was graded as follows: negative: 0; weak signal: 1; middle: 2; and strong: 3. Then the total score resulting from the multiple value of positive percentage score and strength score is as follows: 0-1: grades negative (-); 2-4: weak positive (+); 5-8: middle (++); and 9-12: strong (+++).

### 2.11. Statistical Analysis

All experiments were repeated at least three times, and the results are presented as the mean ± SD. Analyses of significance were performed using Student's* t*-tests or one-way ANOVAs, followed by Bonferroni corrections.* P* < 0.05 was considered statistically significant.

## 3. Results

### 3.1. URG11 Is Expressed Human Prostate Specimens and in Cancer Cells

**Figure 1:**
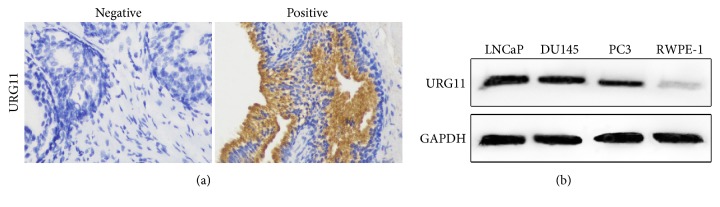
**URG11 expression is upregulated in prostate cancer tissues and in cell lines.** (a) Tissue samples from 68 cases of prostate cancer and 74 cases of benign prostatic hyperplasia were subjected to immunohistochemistry with the antibody of URG11. Representative images of negative and positive URG11 staining were shown. (b) Cell culture of prostate cancer cell lines DU145, LNCaP, and PC3 and nontumor human prostate epithelial cells RWPE-1 was subjected to western blot, with the antibody against URG11.

We first investigated the expression levels of URG11 in clinical prostate cancer specimens. Surgical specimens from 68 cases of prostate cancer and 74 cases of benign prostatic hyperplasia collected from the First Affiliated Hospital of Jinan University and Sun Yat-sen University Cancer Center were immunohistochemically stained for URG11. As shown in Figure 1(a), URG11 protein exhibited cytoplasmic distribution. The expression levels of URG11 in these specimens were shown in Table 1. Positive expression ratio in prostate cancer group (PCa) was 70.6% (48/68) and that in benign prostatic hyperplasia (BPH) group was 21.6% (16/74), suggesting that URG11 levels were significantly upregulated in prostate cancer tissues (*p* < 0.01). Correlations between URG11 expression levels and clinic-pathological parameters are presented in Table 2. The expression levels of URG11 in higher grades (G2 and G3) showed stronger staining than low grade (G1) (*p* < 0.05). The URG11 levels in progressive TNM stages (III + IV stages) showed higher expressions than that in localized stages (I + II stages) (*p* < 0.05). The expression levels in metastatic prostate cancer showed higher staining signals than that in nonmetastatic cancer (*p* < 0.05). There was statistically significant correlation between URG11 expression level and histologic grade (Table 3, r = 0.354,* p* < 0.05); also there was positive correlation between URG11 expression level and TNM stage (Table 4, r = 0.74,* p* < 0.05). These data suggest that URG11 is highly expressed in prostate cancer samples and positively correlated with histologic grade and TNM stage. Further, we determined the expression levels in prostate cancer cells, including DU145, LNCa, and PC3 cell, and nontumor human prostate epithelial cells (RWPE-1). As shown in Figure 1(b), the URG11 levels in human prostate cells were significantly higher than in epithelial cells. Together, these results reveal that URG11 may play an important role in the development and metastasis in human prostate cancer.

### 3.2. Genetic Knockdown of URG11 Suppresses Cell Growth, Migration, and Invasion of Cultured Prostate Cancer Cells

**Figure 2:**
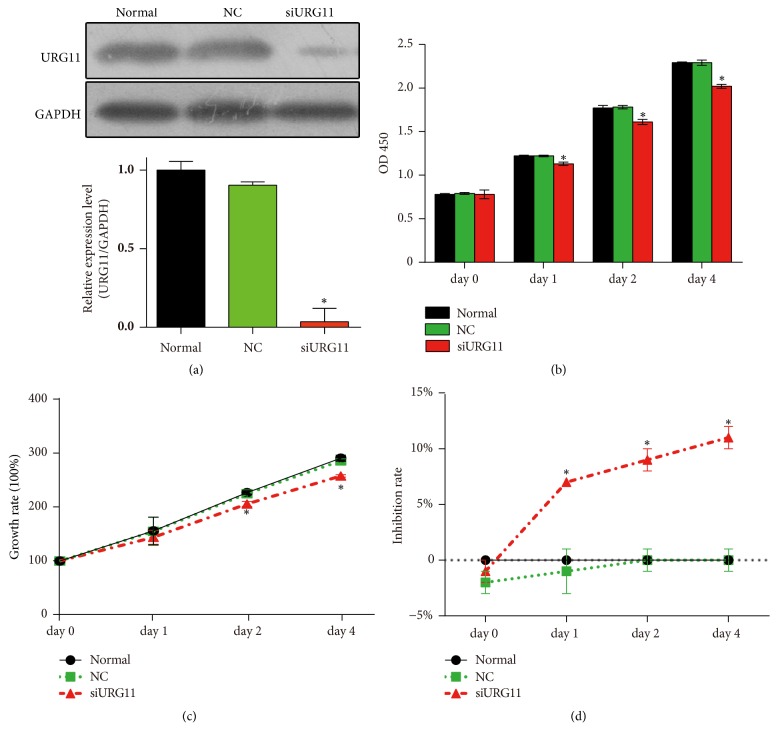
**Genetic knockdown of URG11 suppresses the proliferation and growth of prostate cancer cells.** (a) Cultured LNCaP cells were transfected with siRNA fragments of NC (control) or against URG11 for 48 h. Then cells were harvested and subjected to western blot using URG11 antibody. Representative images were shown in the up panel; the statistical data of relative expression level compared to normal culture were shown in the lower panel. To determine the impact of URG11 knockdown on cell proliferation, LNCaP cells were cultured up to 4 days and cells were harvested at indicted time points for each group. Cell lysate was subjected to MTS. (b) OD 450 values, (c) growth rate, and (d) inhibition rate in each group were shown. All experiments were performed in triplicate, and results are expressed as the mean ± SD. *∗* denotes* p* < 0.05 versus NC control group.

**Figure 3:**
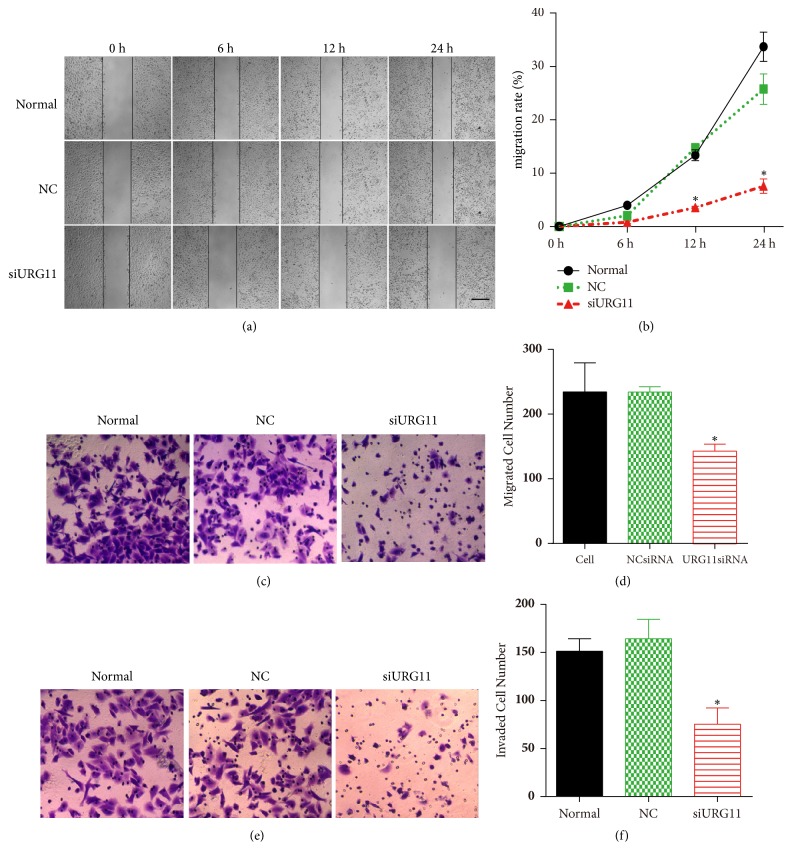
**Inhibition of URG11 impairs migration and invasion of prostate cancer cells.** Cultured LNCaP cells were subjected to wound healing assay (a, b), Transwell migration, (c, d) and invasion (e, f) assay. Representative images in each group were shown and the data were counted from triple experiments and presented as the mean ± SD. *∗* denotes* p* < 0.05 versus NC control group.

To further explore the role of URG11 in prostate cancer cells, we applied siRNA approach to silence URG11. Accordingly, we synthesized siRNA fragments against URG11 which had been proved functional [12]. On the transfection of siRNA fragments into LNCaP prostate cells (siURG11 group), the expression level of URG11 was significantly suppressed (Figure 2(a)) and GAPDH levels was used as loading control. By determining the effective knockdown of the siRNA fragments, we applied MTS assay to reveal the effect of URG11 silencing on cell growth. Cells were cultured for up to 4 days and were harvest for analysis in each day. As shown in Figure 2(b), cells in the URG11 siRNA group showed decreased growth compared to normal culture and NC group (control group). The statistical data of growth rate in different groups were shown in Figure 2(c) and the inhibition rate was shown in Figure 2(d). These data show that inhibition of URG11 expression significantly suppresses cell growth in prostate cancer cells. Moreover, cell migration and invasion were revealed by wound healing assay and Transwell assay. Wounds generated on cells in siURG11 group did not heal for 24 h, at which time wounds in normal culture and in control group were much more healed (Figure 3(a)). The statistical data were shown as Figure 3(b). Furthermore, migration and invasion experiments by Transwell assay revealed that genetic knockdown of URG11 significantly suppressed migration and invasion of LNCaP prostate cancer cells compared with those of normal culture and NC control group (Figures 3(c)–3(f)). Collectively, these data suggest that URG11 is critical for the growth, migration, and invasion of prostate cancer cells.

### 3.3. URG11 Silencing Induces Cell Cycle Arrest and Apoptosis and Decreases *β*-Catenin Expression in Prostate Cancer Cells

**Figure 4:**
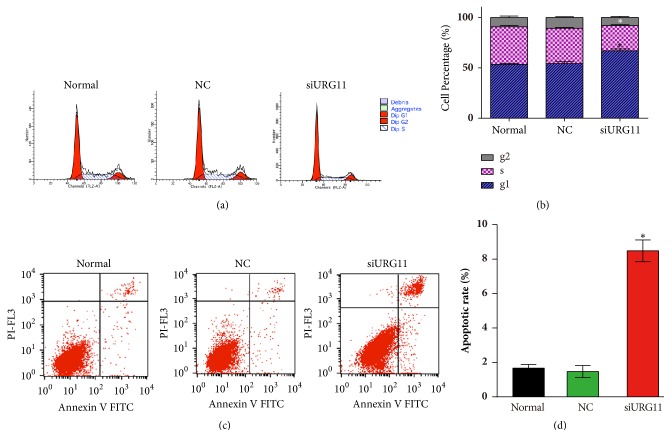
**Inhibition of URG11 induces cell cycle arrest and apoptosis of prostate cancer cells. **Cultured LNCaP cells were subjected to flow cytometry assay using PI staining to determine cell cycle (a, b) and using annexin V and PI staining to determine apoptosis (c, d) in each group. Representative images in each group were shown and the data were counted from triple experiments and presented as the mean ± SD. *∗* denotes* p* < 0.05 versus NC control group.

**Figure 5:**
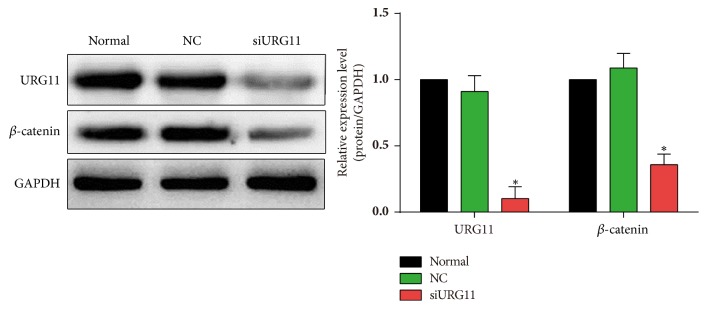
**Inhibition of URG11 decreases the level of** **β** **-catenin. **Cultured LNCaP cells under the different treatment as in Figure 2 were subjected to western blot. Antibodies against URG11 and *β*-catenin were stained for representative show. GAPDH was used as loading control.

To better understand the role of URG11 in prostate cancer metastasis and the detailed mechanism, flow cytometric analysis was performed with PI- and/or annexin V staining for apoptosis and cell cycle analysis. URG11 knockdown showed significant cell cycle arrest, compared with that in normal culture and in NC control group (Figures 4(a)-4(b)). URG11 silencing markedly increased the number of cells in G1 phase and decreased in S and G2/M phase (*p* < 0.05). Further, by annexin V and PI staining under flow cytometric analysis, URG11 silencing significantly induced cell apoptosis, compared with those in normal culture and NC group (Figures 4(c)-4(d)). Moreover, we tried to explore the detailed signaling pathway that might be involved in URG11 regulated prostate cancer cell development. Cells transfected with URG11 fragments were harvested and subjected for western blot. As shown in Figure 5, URG11 silencing significantly suppressed the expression level of *β*-catenin. Together, these data suggest that URG11 is involved in cell cycle progression and inhibition of URG11 would induce cell cycle arrest and cell apoptosis, where the molecular signaling pathway via *β*-catenin may be involved.

### 3.4. Overexpression of URG11 Promotes the Growth, Migration, and Invasion of Prostate Cancer Cells

**Figure 6:**
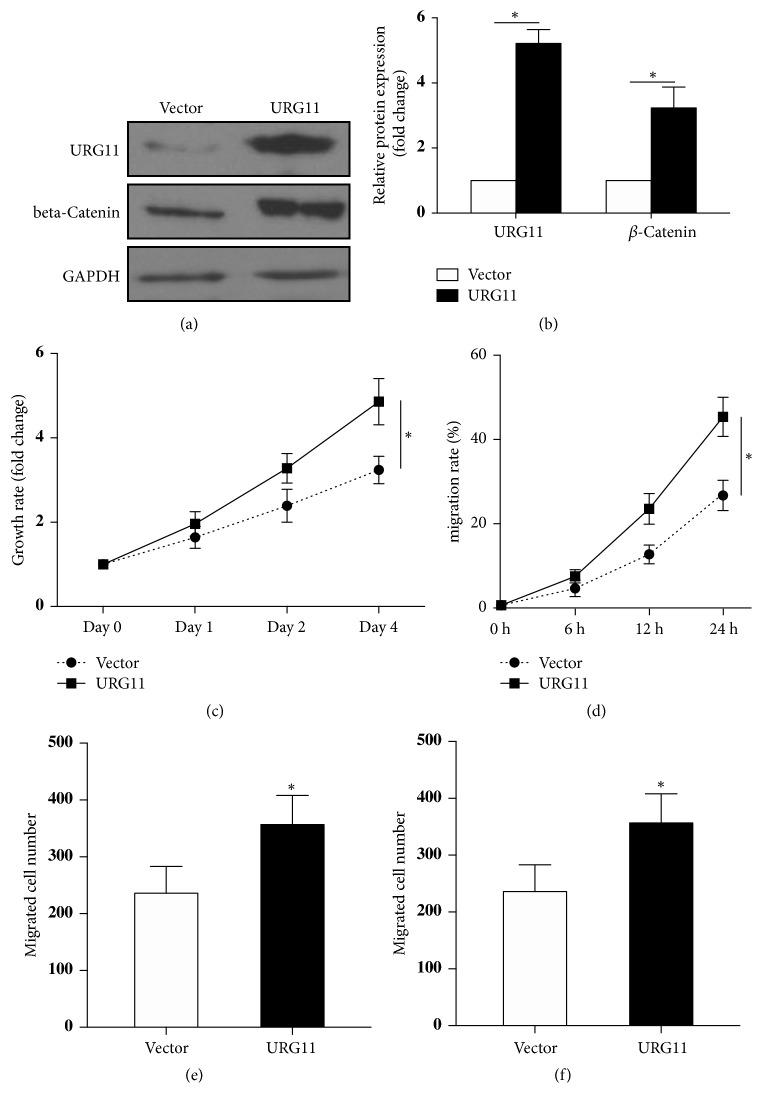
**Overexpression of URG11 increases the growth, migration, and invasion of prostate cancer cells.** URG11 overexpression plasmid was constructed and transfected into LNCaP cells. The protein expression levels were determined by western blot (a) and the statistical analysis of relative fold changes was shown in (b). The growth rate of LNCaP cells was determined by MTS assay (c) and the proliferation rate was determined by wound healing assay (d). Transwell system was applied to test the migration and invasion ability of URG11 overexpressed cells and the data were shown in (e) and (f). Mean ± SD. *∗* denotes* p* < 0.05 versus Vector control group.

Above data showed that inhibition of URG11 markedly suppressed cell proliferation, migration, and invasion of prostate cells. Then we asked whether overexpression of URG11 would result in the opposite. We constructed an URG11 encoding plasmid and overexpressed the plasmid in LNCaP prostate cancer cells. The expression of URG11 was confirmed by western blot and the results revealed the upregulation of *β*-catenin along with URG11 overexpression (Figure 6(a)). The statistical data were shown in Figure 6(b). Prostate cancer cells with URG11 overexpressed showed increased cell proliferation by MTS assay (Figure 6(c)) and by wound healing assay (Figure 6(d)). Furthermore, overexpression of URG11 significantly promoted the migration and invasion of LNCaP cells (Figures 6(e) and 6(f)). These data indicate that overexpression of URG11 could promote the growth, migration, and invasion of prostate cancer cells.

## 4. Discussion

URG11, as an effector of hepatitis B virus X protein, is upregulated in a variety of human cancers, including hepatocellular cancer, gastric cancer, colon cancer, lung cancer, esophageal cancer, and breast cancer [7]. URG11 plays an important role in the development and metastasis in these cancers [12]. However, the role of URG11 in prostate cancer remains to be elucidated. Consistently, in the current study, our data demonstrated that URG11 levels were significantly more upregulated in clinical human prostate cancer tissues than in benign prostatic hyperplasia control tissues. The expression levels of URG11 were closely correlated with histologic grade and TNM stage. Also, URG11 expressions were more increased in prostate cancer cell lines than nontumor human prostate epithelial cells. Genetic knockdown of URG11 markedly suppressed proliferation, migration, and invasion, induced cell cycle arrest and apoptosis, and decreased the level of *β*-catenin in prostate cancer cells. Overexpression of URG11 significantly increased the growth, migration, and invasion of prostate cancer cells. Taken together, these data indicate that URG11 may function as an oncogene in prostate cancer. URG11 may be involved in the early formation and development of prostate cancer, of which the early stage is androgen dependent.

Aberrantly expression of URG11 is critical in carcinogenesis. According to the literature, silencing URG11 suppressed the proliferation of hepatocellular carcinoma cells via downregulation of several G1/S phase-related proteins and attenuates hepatocellular tumor growth in nude mice [16]. By genetic knockdown of URG11, proliferation was inhibited and the invasion was suppressed in pancreatic cancer cells [10, 11]. Consistent with previous findings, our data also found that knockdown of URG11 significantly inhibited proliferation, migration, and invasion of prostate cancer cells. All of these data suggest that URG11 may provide a new way to explore the molecular mechanisms of prostate cancer.

Interestingly, we found that *β*-catenin level was potently decreased upon URG11 knockdown and increased with URG11 overexpression. *β*-catenin is the main structure of adhesion between cells and is necessary for the creation and maintenance of epithelial cell layer. *β*-catenin is a critical end component in the Wnt signaling pathway, which is involved in promoting tumor progression, regulation of cell growth, proliferation, and invasion [17–19]. Wnt/*β*-catenin pathway controls the expression of many downstream target genes such as cyclin D1, matrix metalloprotease-7, and c-Myc, leading to uncontrolled cell growth and invasion [20–23]. Accumulating studies have indicated that aberrantly activation of Wnt/*β*-catenin pathway is implicated in prostate cancer tumorigenesis [24–27]. URG11 has been reported to be the upstream activator to promote tumor growth; inhibition of URG11 decreased the expression level of *β*-catenin and the downstream targets including c-Myc, cyclin D1, and MMPs [11, 28]. Consistent with the reports, we also found that inhibition of URG11 would decrease the level of *β*-catenin, suggesting the same inactivation of Wnt/*β*-catenin signaling pathway. Detailed exploring of URG11 regulated *β*-catenin would be performed in our further study, and role of URG11 would be determined in animal model* in vivo*.

Taken together, the study here provides evidence that URG11 is positively correlated to prostate cancer tumorigenesis and metastasis, inhibition of URG11 suppresses proliferation, migration/invasion, and *β*-catenin levels in prostate cancer cells. URG11 might be a potential novel clinical target for prostate cancer.

## Figures and Tables

**Table 1:** **Expression of URG11 in tissues of prostate cancer (PCa) and benign prostatic hyperplasia (BPH)**.

Group	Case No.	URG11 expression level				*χ*2	*P *value
		(-)	(+)	(++)	(+++)		
PCa	68	20	17	17	14	44.69	0.002
BPH	74	58	16	0	0		

**Table 2:** Relationship of URG11 expression and clinic-pathological parameters in prostate cancer specimens.

Clinicopathologic parameters	Case No.	URG11 expression level				*χ*2	*P *value
		(-)	(+)	(++)	(+++)		
Age						0.002	0.968
<65	42	7	6	6	5		
≥65	44	13	11	11	9		
Histologic grade						9.920	0.007
G1	17	9	5	3	0	G1 vs. G2	0.011
G2	28	7	6	9	6	G1 vs. G3	0.003
G3	23	4	6	5	8	G2 vs. G3	0.440
TNM stage						9.348	0.002
I + II	22	12	4	5	1		
III + IV	46	8	13	12	13		
Metastasis						5..567	0.018
No	27	12	6	7	2		
Yes	41	8	11	10	12		

**Table 3:** Correlation of URG11 expression and histologic grade.

Histologic grade	Case No.	URG11 expression level				*r* value	*P* value
		(-)	(+)	(++)	(+++)		
G1	17	9	5	3	0	0.354	0.003
G2	28	7	6	9	6		
G3	23	4	6	5	8		

**Table 4:** Correlation of URG11 expression and TNM stage.

TNM stage	Case No.	URG11 expression level				*r* value	*P* value
		(-)	(+)	(++)	(+++)		
I + II	22	12	4	5	1	0.740	0.002
III + IV	46	8	13	12	13		
